# Severe Antiphospholipid Syndrome and Diffuse Glomerulonephritis After Adalimumab Treatment in a Patient With Ulcerative Colitis

**DOI:** 10.1155/2024/8024757

**Published:** 2024-11-04

**Authors:** Ileana Rivera-Burgos, Luis M. Vilá

**Affiliations:** Division of Rheumatology, University of Puerto Rico, Medical Sciences Campus, San Juan, Puerto Rico, USA

**Keywords:** adalimumab, antiphospholipid syndrome, glomerulonephritis, tumor necrosis factor alpha inhibitors, ulcerative colitis

## Abstract

Tumor necrosis factor alpha inhibitors (TNFi) are biological drugs used worldwide to treat various autoimmune disorders. Paradoxically, TNF-*α* antagonists can also induce autoimmune diseases being systemic vasculitis, systemic lupus erythematosus, and psoriasis, the most common. We present a 22-year-old woman with ulcerative colitis (UC) who was started on adalimumab 40 mg subcutaneously every 2 weeks. After two doses of adalimumab, she developed gangrene of all toes and acute kidney injury requiring hemodialysis. Skin biopsy showed thrombi in the small vessels of the dermis. Renal biopsy disclosed diffuse proliferative glomerulonephritis (GN) and acute tubulointerstitial nephritis. Serologic work-up showed positive IgG anticardiolipin (ACL) antibodies and low C3 levels. Antinuclear, anti-dsDNA, anti-Smith, anti-SSA, anti-SSB, anti-RNP, antineutrophil cytoplasmic antibodies, ACL (IgA and IgM), and anti-*β*2-glycoprotein I (IgG, IgM, and IgA) antibodies were not elevated. Lupus anticoagulant test and cryoglobulins were negative. Adalimumab was discontinued, and she was treated with enoxaparin, intravenous (IV) methylprednisolone pulse, IV cyclophosphamide, and plasmapheresis followed by maintenance therapy with warfarin, prednisone, azathioprine, and hydroxychloroquine. She did not have further thrombotic events, and the acute kidney injury completely resolved. ACL IgG antibodies decreased to normal levels, and repeated tests were negative. After 7 years, anticoagulation and immunosuppressive drugs were discontinued. During a follow-up of 24 months, she remained in complete clinical remission. This report highlights the occurrence of autoimmune disorders induced by TNFi. Thus, careful monitoring of adverse immune reactions to TNFi is highly recommended.

## 1. Introduction

Tumor necrosis factor alpha inhibitors (TNFi) are biological drugs used worldwide to treat autoimmune disorders such as inflammatory bowel disease (IBD), rheumatoid arthritis, axial spondyloarthropathies, and juvenile idiopathic arthritis [1]. TNFi are highly clinically effective and usually well tolerated [2]. Common side effects include pain at the injection site, headaches, and rashes [3]. Less frequently, TNFi may also cause serious adverse events such as severe infections and paradoxical induction of autoimmune disorders [4]. Among drug-induced autoimmune phenomena are vasculitis, systemic lupus erythematosus, psoriasis, interstitial lung disease, antiphospholipid syndrome (APS), sarcoidosis, uveitis, and inflammatory myopathies [5]. However, severe APS and glomerulonephritis (GN) are rare complications. Herein, we present a case of a young woman with ulcerative colitis (UC) who developed severe APS and acute kidney injury after starting therapy with adalimumab.

## 2. Case Presentation

A 22-year-old woman with a history of UC, APS, and GN treated with prednisone, mesalamine, warfarin, azathioprine, and hydroxychloroquine was hospitalized because of loss of consciousness, abdominal pain, vomiting, profuse hematochezia, and severe anemia [6].

**Figure 1:**
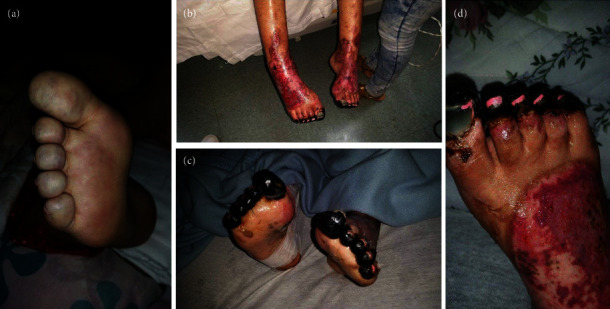
Images of distal lower extremities: (a) right foot with light blue and mottling discoloration, (b) acute ischemic changes of the legs and feet, (c) bilateral digital gangrene of all toes, and (d) right foot digital gangrene with dorsal superficial ulcerations.

**Figure 2:**
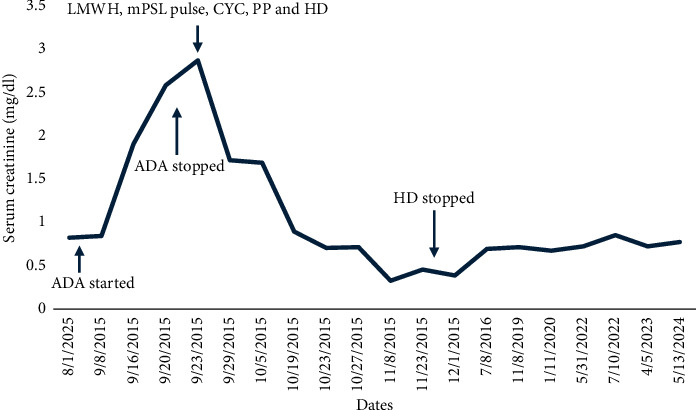
Trend of serum creatinine levels before and after discontinuation of adalimumab (ADA). CYC, cyclophosphamide; HD, hemodialysis; mPSL, methylprednisolone; PP, plasmapheresis.

Seven years before hospitalization, she was diagnosed with UC manifested by chronic colicky abdominal pain, nonbloody diarrhea, rectosigmoid inflammation (erythema, friability, and erosions on colonoscopy), and colon biopsy showing cellular infiltrate of lamina propria, lymphoid aggregates, and distortion of crypt architecture. She was treated with high-dose intravenous (IV) methylprednisolone followed by oral prednisone, but because of recurrent disease, she was started on adalimumab 40 mg subcutaneously every 2 weeks. After two doses of adalimumab, she developed cyanosis of all toes that rapidly progressed to gangrene in less than 1 week (Figure 1). Histological examination of deeper sections of the dermis of the right third toe disclosed thrombi in most small vessels. Immunohistochemistry tests to determine the extent of inflammatory cell infiltration (CD45), lymphovascular invasion (D2-40), and expression of monocytes/macrophages (CD68) were negative. Vasculitis was not observed. Also, she had an acute kidney injury that required hemodialysis. Urine analysis showed proteinuria, hematuria, and pyuria. Renal biopsy disclosed diffuse proliferative GN and acute tubulointerstitial nephritis. There was mild to moderate polymorphonuclear infiltration of the glomeruli, which were hypercellular with endocapillary cell proliferation and obliteration of the capillary lumina. The interstitium showed mild to moderate lymphoplasmacytic infiltration with tubulitis. Direct immunofluorescence showed deposition of IgM (+2; segmental and granular pattern in glomerular basement membranes) and C3 (+4; global and garland pattern in glomerular basement membranes). Immunofluorescence staining for IgA, IgG, C1q, fibrinogen, kappa chain, and lambda chain was negative. There was no significant fibrosis or tubular atrophy, and the vasculature showed no fibrosis or vasculitis. No thrombotic microangiopathy lesions were observed. Serologic work-up showed moderate elevation of IgG anticardiolipin (ACL) antibodies (58 GPL) and low C3 levels at 30.5 mg/dL (normal range 90–180 mg/dL). Antinuclear antibodies, anti-dsDNA, anti-Smith, anti-RNP, and Ro, ant-La, anti-beta-2 glycoprotein 1 (IgA, IgG, and IgM), and IgA and IgM ACL antibodies were negative. C4 level was normal, and lupus anticoagulant and cryoglobulin tests were negative. She had no other manifestations suggestive of systemic lupus erythematosus or other autoimmune rheumatic disorders. She had no coexistent infections, and urine and blood cultures were negative. She had no laboratory features suggestive of disseminated intravascular coagulation such as thrombocytopenia, prolonged partial prothrombin time or partial prothrombin time, low fibrinogen level, or high plasma D-dimer level. Also, work-up for other hypercoagulable disorders, such as protein C, protein S, and antithrombin III deficiencies, was negative. Adalimumab was discontinued, and she was treated with enoxaparin 60 mg subcutaneously every 12 h, pulse methylprednisolone (1 g IV daily for 3 days) followed by high-dose methylprednisolone (1 mg/kg/day), IV cyclophosphamide pulse (1 g, single dose), and plasmapheresis (for three consecutive days). She responded well to this therapy. She did not develop further thrombotic events, and GN completely resolved after 3 months. The trend of serum creatinine levels before and after discontinuation of adalimumab is shown in Figure 2. She was started on maintenance therapy with warfarin, prednisone, azathioprine, and hydroxychloroquine. C3 levels normalized, and IgG ACL antibodies decreased to normal levels. Afterward, repeated serologic tests were consistently negative. On the other hand, her UC remained persistently active, having multiple hospitalizations and treatments with high-dose corticosteroids, and requiring left-side partial colectomy. Also, she failed vedolizumab treatment.

On admission, she presented with tachycardia of (140 beats per minute) and hypotension (98/50 mm Hg). Her physical examination showed a distended and diffusely tender abdomen with positive guarding and rebound tenderness. Her lower extremities demonstrated amputated toes. The rest of the physical examination was unremarkable. Blood cell count showed leukocytosis at 15,800/*μ*l, and hypochromic microcytic anemia with hemoglobin at 6.3 g/dL. The international normalized ratio was markedly elevated at 8.0. Serum chemistry tests and urine analysis were normal. Complete serologic testing including C3 and C4 levels, lupus anticoagulant test, and antinuclear, anti-dsDNA, anti-Smith, anti-RNP, Anti-Ro, anti-La, ACL (IgA, IgG, and IgM), and anti-beta-2 glycoprotein 1(IgA, IgG, and IgM) antibodies were negative. The abdominopelvic computed tomography showed findings consistent with active inflammation of the descending colon, sigmoid colon, and rectum. In view of severe colitis and persistent lower gastrointestinal bleeding, she underwent total colectomy with end ileostomy and mucous fistula creation.

After an extensive investigation and review of her clinical course, it was concluded that APS and GN were associated with the use of adalimumab. Thus, warfarin, azathioprine, and hydroxychloroquine were discontinued. Low-dose aspirin was started, and prednisone dose was gradually decreased and discontinued after 6 months. After a 24-month follow-up, she remained in clinical remission without developing renal abnormalities or thrombotic events.

## 3. Discussion

We report a case of severe APS and GN/interstitial nephritis associated with adalimumab exposure. APS was characterized by gangrene of distal toes bilaterally and elevated ACL IgG antibodies. Renal disease was documented by biopsy which showed diffuse proliferative GN and acute tubulointerstitial nephritis. She responded well to anticoagulation, immunosuppressive treatment, and discontinuation of adalimumab. Eventually, ACL antibodies became negative, and anticoagulation and immunosuppressive treatment was discontinued without resulting in recurrent thrombotic events or renal disorders.

Since the approval of TNFi by the US Food and Drug Administration (FDA) in 1998, these agents have revolutionized the treatment of multiple autoimmune conditions such as UC, Crohn's disease, rheumatoid arthritis, psoriasis, psoriatic arthritis, ankylosing spondylitis, and juvenile idiopathic arthritis [7]. There are five FDA-approved TNFi: etanercept, infliximab, adalimumab, golimumab, and certolizumab pegol [8]. Although these agents have been extensively studied, several investigations have shown a paradoxical occurrence of autoimmune phenomena ranging from asymptomatic immunologic alterations to life-threatening systemic autoimmune diseases [9, 10]. TNFi have shown to induce antinuclear antibodies, anti-dsDNA antibodies, and antiphospholipid antibodies, among others [11]. Several autoimmune diseases have been associated with TNFi exposure, being vasculitis, systemic lupus erythematosus, and psoriasis, the most frequently reported [12]. Although the pathogenesis of these immune adverse events is unknown, some studies have suggested the potential role of interferon-*α* (IFN-*α*) in the induction of these disorders [13]. TNF*α* inhibits the production of IFN*α* from peripheral dendritic cells; thus, inhibition of TNF may lead to increased production of IFN-*α*. This cytokine plays a crucial role in the immunopathogenesis of several disorders, including SLE, IBD, and psoriasis [14].

APS is an acquired autoimmune disease characterized by thromboembolic events or pregnancy morbidity in association with the presence of antiphospholipid antibodies [15]. The most feared complication of APS is catastrophic APS [16]. Although our patient did not meet the classification criteria of catastrophic APS, she had multiple digital ischemia and gangrene that warranted aggressive treatment [17]. According to the BIOGEAS registry, a multicenter study aimed at collecting data on biologic agents in adults with autoimmune disorders, 32 cases with APS or APS-like features associated with TNF*α* blockers were reported [18]. Of these cases, the most common manifestations were venous thrombotic disorders such as deep venous thrombosis, thrombophlebitis, and pulmonary embolism. In contrast to our patient, no arterial thrombotic events were reported in this registry.

In addition to the data reported by the BIOGEAS registry on the association of APS and TNFi, similar clinical scenarios have been documented on patients treated specifically with adalimumab. For example, Cheemalavagu, McCoy, and Knight reported a 50-year-old woman with Crohn's disease treated with adalimumab for 14 months who developed arterial thrombosis of the left hand in the presence of positive IgM ACL antibodies and IgM anti-*β*2-glycoprotein I antibodies [19]. Adalimumab was stopped, and she was treated with full anticoagulation. Antiphospholipid antibodies became negative 20 weeks later. Warfarin was discontinued, and she remained free of thrombotic events after 3 years of follow-up. Most recently, Uehara et al. reported a patient with Crohn's disease treated with adalimumab who developed APS manifested by pulmonary embolism and antiphospholipid antibodies [20]. The patient was treated with anticoagulation, and adalimumab was discontinued. As in the case reported by Cheemalavagu et al. and ours, antiphospholipid antibodies became negative, and the patient did not have recurrent thrombotic events.

It is important to highlight that patients with IBD are at higher risk for arterial and venous thrombotic events; this could have further increased the risk for thrombosis in our patient [21, 22]. Although the pathophysiology is not clearly defined, the hypercoagulable state in IBD seems to be related to the inflammatory process. Venous thrombosis, mainly deep venous thrombosis of lower extremities and pulmonary embolism, is more frequent than arterial thrombosis. Risk factors of thrombotic events include active disease, hospitalizations, and postoperative periods. Furthermore, patients with IBD, particularly those with Crohn's disease, have a higher prevalence of antiphospholipid antibodies [23]. In patients with Crohn's disease, ACL antibodies and anti–*β*2-glycoprotein I antibodies have been reported in 23.4% and 9.7%, respectively, whereas 4.8% and 9.7% have been reported for UC patients. In IBD, ACL antibodies and anti–*β*2-GPI antibodies usually remain stable over time [23]. This behavior contrasts with our patient in which ACL antibodies normalized after discontinuation of adalimumab.

Our patient also presented with severe acute GN and tubulointerstitial nephritis requiring hemodialysis. The association of acute kidney injury in association with TNFi is not unexpected. In 2017, the BIOGEAS registry published a review of 12,731 cases of autoimmune diseases induced by biologic agents [24]. The registry reported 25 cases of GN induced by TNFi, including eight cases of adalimumab. Also, five cases of interstitial nephritis were reported, mainly after treatment with infliximab. Furthermore, four patients with axial spondyloarthropathy treated with adalimumab-developed acute kidney injury [18]. In all cases, adalimumab was stopped, and all received systemic corticosteroids. As in our case, all patients clinically improved after TNFi discontinuation.

Another diagnostic consideration in this case is drug-induced thrombotic microangiopathy (DITMA), characterized by a broad spectrum of clinical manifestations including microangiopathic hemolytic anemia, thrombocytopenia, acute kidney injury, central nervous system involvement, and gastrointestinal and skin manifestations [25]. Various drugs, such as chemotherapeutic agents, targeted cancer therapies, antibiotics, antivirals, and immunosuppressants, have been linked to DITMA. Specifically, DITMA secondary to TNFi has been documented. For instance, Falsetti et al. reported on a 63-year-old man with Crohn's disease who developed confusion, severe microangiopathic hemolytic anemia, thrombocytopenia, and leukopenia after a single dose of adalimumab [26]. Adalimumab was discontinued, and the patient underwent plasma exchange, leading to complete recovery. Similarly, Baysal et al. described a 39-year-old man with ankylosing spondylitis who, after 6 months of certolizumab pegol treatment, developed intravascular hemolytic anemia and thrombocytopenia [27]. Discontinuation of certolizumab pegol and treatment with systemic corticosteroids and plasma exchange resulted in significant clinical improvement. In our patient, skin biopsy revealed thrombi within the lumina of small dermal blood vessels without evidence of vasculitis, which is characteristic of DITMA. However, she did not present with hematologic abnormalities, and kidney biopsy did not show thrombi. Therefore, the diagnosis of DITMA is less likely in our case. Also, the likelihood of primary (non-drug–induced) autoimmune rheumatic diseases in our patient is low, as she did not experience recurrent symptoms after discontinuing all immunosuppressive drugs.

To the best of our knowledge, we present the first case of concurrent severe APS and acute kidney injury associated with TNFi. As reported in other cases, withdrawal of TNFi and treatment of associated complications resulted in a favorable clinical response. TNFi are increasingly used due to their clinical efficacy and broad spectrum of medical indications; thus, an increase in related immune adverse events is also expected. Physicians should be aware of the paradoxical induction of autoimmune disorders by TNFi. Failure in prompt recognition and withdrawal of the offending biologic agent may result in a poor clinical outcome and irreversible disease damage.

## Data Availability

The data used to support the findings of this study are available from the corresponding author upon request.
